# Comparison between Injury Severity Score (ISS) and New Injury Severity Score (NISS) in predicting mortality of thoracic trauma in a tertiary hospital

**DOI:** 10.1590/0100-6991e-20243652-en

**Published:** 2024-04-17

**Authors:** MARIANA FRANC GARCIA, RENATO TALES GOMES, EDUARDO CUNHA PUGLIESI, JOAO PAULO VIEIRA DOS SANTOS, FERNANDO DE MARTINO, KAIO HENRIQUE VIANA GOMES, DANILO RODRIGUES GOULART PASQUARELI, ROBERTO DA MATA LENZA

**Affiliations:** 1 - Universidade Federal do Triângulo Mineiro, Medicina - Uberaba - MG - Brasil

**Keywords:** Abbreviated Injury Scale, Injury Severity Score, Thoracic Surgery, Trauma Severity Indices, Thoracotomy, Escala de Gravidade do Ferimento, Brasil, Cirurgia Torácica, Traumatismos Torácicos, Tórax

## Abstract

**Introduction:**

measuring the severity of traumatic injuries is crucial for predicting clinical outcomes. Whereas the Injury Severity Score (ISS) has limitations in assigning scores to injuries at the same site, the New Injury Severity Score (NISS) corrects for this problem by taking into account the three most severe injuries regardless of the region of the body. This study seeks to comprehend the clinical and epidemiological profile of trauma patients while comparing the effectiveness of scales for predicting mortality.

**Methods:**

a descriptive, observational and retrospective study using records of patients who underwent thoracotomy at the Hospital das Clínicas of the Federal University of Triângulo Mineiro between 2000 and 2019. Demographic data, mechanisms of injury, affected organs, length of stay and mortality were analyzed. Injury severity was assessed using the ISS and NISS, and statistical analyses were conducted using MedCalc and SigmaPlot.

**Results:**

101 patients were assessed, on average 29.6 years old, 86.13% of whom were men. The average duration of hospitalization was 10.9 days and the mortality rate was 28.7%. The ROC curve analysis revealed a sensitivity of 68.97%, specificity of 80.56% and area under the curve of 0.837 for the ISS, and 58.62%, 94.44% and 0.855 for the NISS, respectively. The Youden index was 0.49 for the ISS and 0.53 for the NISS.

**Conclusion:**

the study demonstrated comparable efficacy of NISS and ISS in predicting mortality. These findings hold significance in the hospital setting. Professionals must be familiar with these scales to utilize them competently for each patient.

## RESUMO

**Introdução:**

a medição da gravidade das lesões traumáticas é essencial para prever os desfechos clínicos. Enquanto o Injury Severity Score (ISS) tem limitações ao atribuir pontuações às lesões no mesmo local, o New Injury Severity Score (NISS) corrige esse problema ao considerar as três lesões mais graves independentemente da região corporal. Este estudo visa entender o perfil clínico-epidemiológico dos pacientes traumatizados, comparando a eficácia das escalas para prever mortalidade.

**Métodos:**

estudo descritivo, observacional e retrospectivo utilizando registros de pacientes submetidos à toracotomia no Hospital das Clínicas da Universidade Federal do Triângulo Mineiro entre 2000 e 2019. Dados demográficos, mecanismos de lesão, órgãos afetados, tempo de internação e mortalidade foram analisados. A gravidade das lesões foi avaliada usando o ISS e NISS, e as análises estatísticas foram conduzidas no MedCalc e SigmaPlot.

**Resultados:**

Foram avaliados 101 pacientes, em média com 29,6 anos, sendo 86,13% homens. A média da internação foi de 10,9 dias e a taxa de mortalidade foi de 28,7%. A análise da curva ROC revelou uma sensibilidade de 68,97%, especificidade de 80,56% e área sob a curva de 0,837 para o ISS, e 58,62%, 94,44% e 0,855 para o NISS, respectivamente. O índice de Youden indicou 0,49 para o ISS e 0,53 para o NISS.

**Conclusão:**

o estudo demonstrou semelhante eficácia entre o NISS e o ISS na previsão de mortalidade. Esses resultados geram implicações importantes na aplicação dessas escalas no ambiente hospitalar. É essencial que os profissionais conheçam tais escalas para aplica-las adequadamente no contexto de cada paciente.

## INTRODUCTION

Trauma is an injury caused by the exchange of energy between the environment and the body, resulting in injuries to different systems and organs^1^, causing significant social, psychological, political, and economic impact. Thoracic trauma results from any injury that affects the chest wall, organs, and/or structures by external forces. It is an important cause of preventable deaths and currently accounts for about 25% of deaths in polytrauma patients^2^. In addition, it is present in 7.3% of external causes of hospitalization, being the second most frequent type of trauma in Brazil^3^. The mechanisms of injury can be divided into blunt and penetrating. Both, when affecting the topography of the chest, generate important consequences, given the intimate relationship between the rib cage and the organs responsible for oxygenation, perfusion, and oxygen flow^2^. The main etiologies of blunt traumas are car accidents, run over, and falls. As for penetrating injuries, they are mostly caused by gunshot and stabbing wounds, with variations according to the territory studied^1^. Injury severity is a determining factor for the outcome of a traumatic event. Scoring systems have been developed with the aim of standardizing severity assessment, by confronting the repercussions of treatments on traumatic injuries.

The Abbreviated Injury Scale (AIS), developed by the Association for the Advancement of Automotive Medicine, corresponds to a global severity scoring system derived from a consensus, which classifies an individual injury by body region according to its relative importance. It is the most widely used instrument for calculating the severity of single injuries, and to assess the overall severity of a patient with multiple injuries, the Injury Severity Score (ISS) and the New Injury Severity Score (NISS) were developed based on the AIS^4^. Since its publication by Baker et al. in 1974, the ISS has been widely disseminated in the classification of victims of blunt and penetrating trauma^5^. To calculate the ISS, the body is divided into six regions: head and neck, face, chest, abdomen, extremities, and external. Each injury in the body is assigned a score based on the abbreviated injury scale (AIS) and only the highest score in each region is counted. Its score ranges from 1 (minimum severity) to 6 (maximum severity) points. If a patient receives a score of 6 in any region, he or she is automatically raised to the maximum final score of 75 points, regardless of the other lesions. The ISS is then calculated by the sum of the squares of the three highest AIS scores^6^. However, as it considers only the most severe injury of each body segment for severity assessment, the ISS has limitations in revealing lesions with lower AIS scores in the same region, even if it obtains greater relevance when compared to injuries of other organs.

To correct these limitations, the NISS was developed. For the calculation of this new score, the three most severe injuries resulting from the traumatic event are considered, regardless of the body region in which they are located^6^. In short, in cases where the main injuries are in different sites, the values of ISS and NISS are the same, but the main difference occurs when the trauma promotes more than one serious injury in a single body region, since the relevant injuries present in the same segment will be accounted for only by the NISS.

In view of the above, this study, carried out at the Hospital das Clínicas of the Federal University of Triângulo Mineiro (HC-UFTM), based on data from 20 years, aims to understand the clinical and epidemiological profile of patients involved exclusively in thoracic trauma and to compare the effectiveness of the ISS and NISS indexes, based on AIS 05 (2008 update), to predict mortality after hospitalization of patients in this tertiary hospital.

## METHODS

This is a descriptive, observational, and retrospective study, based on medical records of trauma victims who underwent thoracotomy between 2000 and 2019 at HC-UFTM. The variables analyzed were sex, age, race, time of occurrence of trauma, time of care at the HC-UFTM Emergency Room, day of the week, trauma mechanism, signs and symptoms at hospital admission, affected organic structures, indication of thoracotomy, surgical interventions performed, length of hospital stay, sequelae, and deaths. All anatomical lesions obtained from the medical records were manually classified according to the AIS-08, establishing trauma severity by calculating the ISS and NISS. The data were tabulated in Excel software for Windows, version 2019 (12527.20482). Two authors checked the application of the scores to correct possible errors. They were grouped into intervals from 1 to 8, 9 to 15, 16 to 24, and 25 to 75 to calculate the frequencies of gravity. The Receiver Operator Characteristic curve was applied using the MedCalc software, version 22.003, in which sensitivity, specificity, and Youden index J were obtained to evaluate the efficacy of the model in predicting mortality given trauma severity. Then, the table with the variables and scores was submitted to statistical tests to evaluate their associations, with a significance level of 95% (p<0.05), using the Sigma Plot 14.0 software. We applied the Pearson’s correlation test between the time of admission to surgery and the length of hospital stay, the chi-square test to compare surgery in the first 12 hours and death, and the Spearman’s correlation test to evaluate the association between the NISS and ISS scores and length of hospital stay, as well as between scores and time elapsed between admission and surgery. The study was previously approved by the Ethics in Research Committee of UFTM under CAAE No. 51746321.0.0000.8667 and Opinion No. 5.136.582. 

## RESULTS

We studied 101 patients, with a mean age of 29.6 years, of whom 86.13% were male. Traumas were more common at night, from 18:00 to 00:00 (44.55%), and on weekends (61%), from Friday to Sunday. The most common mechanism was stabbing wound (48%), followed by gunshot wound (24%), rendering open trauma mechanisms more prevalent (74.25%). Among the most affected regions, the left anterior hemithorax (41.58%) and the right anterior hemithorax (38.61%) stood out, which resulted in the lungs being the most affected organs (53.33%). The length of hospital stay ranged from less than 24 hours to 97 days, with a mean of 10.9. On the other hand, 21.7% of these patients were hospitalized for less than one day, including those who died. The mortality rate of the study was 28.7%. 

As described by Javali et al. (2019)^7^, the present study also used the cut-off points less than 9 (mild), 9 to 15 (moderate), 16 to 24 (severe), and greater than or equal to 25 (profound) for the scores obtained from the ISS score. The classification was used to analyze the values obtained by the ISS and NISS and for comparisons between them.

We found that the greater the severity of the trauma, the greater the discrepancy between these scores.

Of the 101 patients evaluated, both scores classified eight patients as lower than 9, which characterizes them as mild trauma (7.92%). Regarding moderate trauma (9-15 points), the ISS classified 26 patients in this category (25.74%), while NISS, 13 (12.87%). A total of 49 patients were identified as severe trauma by the ISS and 32 by the NISS. In deeply severe traumatic events, described as profound, 18 patients (17.82%) scored more than 25 points on the ISS and 48 (47.52%) on the NISS.

We compared the ISS and NISS scores used to predict mortality with the ROC (Receiver Operator Characteristic) curve. For the ISS (considered the gold standard), the sensitivity was 68.97%, specificity was 80.56%, and the area under the curve was 0.837. Regarding NISS, the sensitivity was 58.62%, specificity was 94.44%, and the area under the curve was 0.855. In both analyses, there was statistical significance, with a value of p<0.0001. Youden’s index showed the best cut-off points of 0.49 and 0.53 for ISS and NISS, respectively. These data are shown in Table 1.

**Table 1:** Analysis of ROC curve data.

ROC CURVE			
Variable	ISS	Variable	NISS
Variable classification	Death	Variable classification	Death
Sample size	101	Sample size	101
Death	29 (28,71%)	Death	29 (28,71%)
Non-death	72 (71,29%)	Non-death	72 (71,29%)
Area under ROC curve	0,837	Area under ROC curve	0,855
Standard Error	0,0415	Standard Error	0,0401
95% confidence interval	0.751 to 0.903	95% confidence interval	0.771 to 0.917
Z Statistic	8,139	Z Statistic	8,843
Significance (p-value) (area=0.5)	<0.0001	Significance (p-value) (area=0.5)	<0.0001
Youden J index	0,4952	Youden J index	0,5307
Association Criteria	>18	Association Criteria	>29 AM
Sensitivity	68,97	Sensitivity	58,62
Specificity	80,56	Specificity	94,44

Source: Data obtained from the analysis of the medical records of trauma patients undergoing thoracotomy

In addition to the analysis covering the ROC curve, we compared the NISS and ISS with different variables, such as time between hospital admission and surgery and length of hospital stay. The analysis that showed a significant association was between NISS and length of hospital stay, assessed with the Spearman’s correlation test, with a coefficient of -0.264 and a p-value of 0.0077. This data reveals that the higher the NISS, the shorter the hospitalization time, a fact mainly related to the patients who died.

We tested other relationships, as shown in Table 2, without statistically significant associations between variables, though. It is important to emphasize that, even with the analysis carried out over 20 years at the HC-UFTM, our sample was small, which may have interfered with results.

**Table 2:** Table of statistical tests performed.

VARIABLES ANALYZED	STATISTICAL TEST	p-VALUE
Time between admission to the service and the start of surgery versus length of hospital stay	Pearson's correlation test	p=0.107
Direct admission to the tertiary hospital versus death	Chi-square test	p=0.962
ISS versus time between admission and surgical intervention	Spearman's correlation test	p=0.979
NISS versus time between admission and surgical intervention	Spearman's correlation test	p=0.501
ISS versus length of hospital stay	Spearman's correlation test	p=0.125
NISS versus length of hospital stay	Spearman's correlation test (-0.264)	p=0.007

Source: Data obtained from the analysis of the medical records of trauma patients submitted to thoracotomy.

[Fig f1]

**Figure 1. f1:**
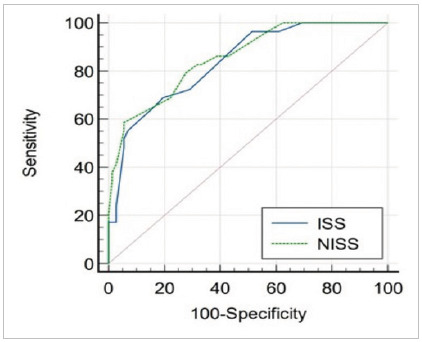

## DISCUSSION

Consistent with what is presented in the literature, the mean age of the individuals studied was 29.6 years. In a range from zero to 79 years, victims between 20 and 29 years of age represent 45.54% of patients, and when analyzing the age group of up to 40 years, the figure increases to 84.15%. These data demonstrate that chest trauma and the classification of its severity are important objects of study, as they deal with a public health problem that mostly affects the economically active population, causing great morbidity and mortality and social impacts in the country^3^. This trend of population involvement relevant to the workforce is also presented in other studies, such as the one by Estrada and Leon (2018), who found that the most affected age group was between 25 and 39 years (37.97%)^8^.

A male prevalence was also demonstrated among the thoracic trauma victims evaluated, totaling 86.13% of the cases. Our data agree with the study by Queiroz et al. (2021), which analyzed the population of the Brazilian state of Sergipe and found a prevalence of 84.2% of male patients among victims of thoracic trauma^9^. This result suggests that men are more prone to traumatic events, a fact justified by sociocultural aspects, resulting in greater aggressiveness in driving vehicles^10^, and an influence of other factors, such as speeding and physical confrontations, associated with alcohol abuse^9^.

As for trauma severity classification, the ISS, introduced in 1974, is the most commonly used score, both because of the professionals’ habit of applying it, and because many studies have not shown any significant difference between the ISS and the NISS in predicting the outcome resulting from trauma^6^, the ISS being considered a kind of “gold standard” by several traumatologists^11^, even with the limitations present in the way it is calculated, which may underestimate the severity of some of these patients. 

In this study, we compared ISS and NISS in the estimation of patient survival in a reference hospital in the Triângulo Mineiro region, when analyzing patients sustaining thoracic trauma and undergoing thoracotomy between 2010 and 2019.

Among the patients analyzed, 73 (72.27%) had NISS values higher than their ISS ones, a figure similar to the result of the one from Domingues et al. (2016), who found 62.9% higher NISS than ISS^12^. This is because ISS considers only the most severely injured site in its calculation and may not include the score of the second most serious site^6^. Thus, in agreement with the literature, when stratifying the scores into mild (<9), moderate (9-15), severe (16-24), and profound (≥25), the difference in scores increases in the most severely injured individuals. A total of 48 patients scored 25 points or more according to the NISS score, i.e., they were classified as profound trauma, while only 18 were classified as such by the ISS. Thus, when analyzing these thoracic trauma victims, it was possible to conclude that the ISS may underestimate the severity of such patients, mainly because it does not account for the various injuries that may be present in the same body region and that could eventually contribute to classifying the patient as a victim of profound trauma. It is important to emphasize that, as the patients evaluated in this study sample were those who underwent thoracotomy, most of them experienced severe thoracic trauma, hence the need for such a surgical approach. 

However, as presented in the literature, this analysis showed no difference between the scores in predicting mortality. The mortality rate found in the sample of patients evaluated was 28.8%. The area under the ROC curve using the ISS and NISS was 0.837 and 0.855, respectively, both scores being statistically significant for mortality prediction.

There are several statistical calculations used in the analysis of the performance of classification models, and one of the most used is the ROC (Receiver Operating Characteristic) curve, which consists of the graphical representation of the performance of a quantitative data model, according to its sensitivity rate and the false positives rate (1-specificity)^13^. Thus, it is an index that is not affected by the threshold effect, making it more effective^11^. The Area Under the Curve (AUC) provides an estimate of the probability of correct classification of a subject at random (test accuracy) and, according to Polo and Miot (2020), AUC values between 0.8-0.9 are interpreted as good^13^, which was the data found in this study.

Regarding the prediction of mortality by scores, Javali et al. (2019) observed no significant difference between the ISS and the NISS, with an AUC of 0.963 for the ISS and 0.970 for the NISS^7^. The study by Li and Ma (2021) compared the two scoring systems in predicting mortality, intensive care unit (ICU) admission, and length of ICU stay, and observed the superiority of the NISS over the ISS in predicting ICU admission and length of stay. However, the prediction of overall mortality of both scores was equivalent, with AUC of 0.886 and 0.887 for ISS and NISS, respectively^6^, i.e., it was classified as “good” by the AUC value, as in this study.

In contrast, other analyses identified in the literature found superiority of NISS over ISS. For example, the study by Bustillo et al. (2018) concluded that the NISS is a more accurate index than the ISS, in addition to having better predictive capacity, with an AUC of 0.811^4^.

Due to the items explored and the purpose of the ISS and NISS in assessing the severity of traumas, several association tests were performed to verify the correlation between some of the variables exemplified below.

Regarding the association between NISS and length of hospital stay, the Spearman’s test showed in an intermediate correlation, with a coefficient of -0.264 (p=0.007), i.e., the higher the NISS, the shorter the patient’s length of stay. This statistically significant relationship is because the higher the NISS value, the greater the severity and complexity of the trauma, and thus there may be a greater possibility of the patient dying in a few hours or days, which consequently leads to a shorter hospitalization time. This relationship between NISS and length of hospital stay has also been demonstrated in other studies. Bustillo et al. (2018) evaluated patients affected by external causes who were admitted to trauma services by emergency room and demonstrated, unlike the present study, that the higher the NISS value, the longer the length of hospital stay, due to the increase in patient severity^4^. Thus, Bustillo discusses the advances in the care of trauma patients, which have resulted in a decrease in mortality, but not necessarily in a reduction in the length of hospital stay or in the resources used^4^. In addition, Ede et al. (2023) evaluated patients with musculoskeletal injuries and compared NISS and ISS scores with length of stay, concluding that NISS has a better classification rate for length of stay^14^. 

The sample size of this study, in which trauma patients and thoracotomy were evaluated at HC-UFTM, a trauma referral center, is a limiting factor. Only 15% to 30% of patients sustaining thoracic trauma require more invasive measures, such as thoracotomy^2^. This restriction, by reducing the sample space and selecting more severely affected patients, may have contributed to some association tests performed resulting in a p-value with low statistical significance. Another limiting condition is the study retrospective nature, i.e., some patients from the sample space were excluded from the analyses due to lack of data.

Despite the limitations and even with an analysis performed in more severe patients, this study showed no statistically significant difference between the ISS and NISS in predicting mortality.

## CONCLUSION

Trauma remains lethal in the population and an alarming issue of public health, mainly affecting young adults and resulting in economic and social security problems, being a significant cause of morbidity, mortality, and disability in the economically active population.

This study demonstrated no difference between the NISS and ISS scores for the prediction of mortality in thoracic trauma. However, the more severe the trauma, the greater the difference between the scores. In patients severely injured in the same region of the body, the ISS may underestimate trauma complexity, while the NISS considers the cumulative effect of multiple injuries in a single region. Thus, studies such as this one are relevant for the dissemination of these scores and their applicability in various hospitals, contexts, and regions. It may allow a greater number of professionals to know the usefulness of such scores, their results, and limitations, and thus use and interpret them according to the context of each patient and institution.
